# Efficacy and safety of zuranolone co-initiated with an antidepressant in adults with major depressive disorder: results from the phase 3 CORAL study

**DOI:** 10.1038/s41386-023-01751-9

**Published:** 2023-10-24

**Authors:** Sagar V. Parikh, Scott T. Aaronson, Sanjay J. Mathew, Gustavo Alva, Charles DeBattista, Stephen Kanes, Robert Lasser, Amy Bullock, Mona Kotecha, JungAh Jung, Fiona Forrestal, Jeff Jonas, Theresa Vera, Bridgette Leclair, James Doherty

**Affiliations:** 1 Department of Psychiatry, University of Michigan, Ann Arbor, MI USA; 2 Institute for Advanced Diagnostics and Therapeutics, Sheppard Pratt, Baltimore, MD USA; 3 Menninger Department of Psychiatry and Behavioral Sciences, Baylor College of Medicine, Houston, TX USA; 4 ATP Clinical Research, Costa Mesa, CA USA; 5 General Psychiatry and Psychology, Stanford University School of Medicine, Stanford, CA USA; 6 EmbarkNeuro, Oakland, CA USA; 7 Sage Therapeutics, Inc., Cambridge, MA USA; 8 Biogen, Cambridge, MA USA

**Keywords:** Drug development, Depression

## Abstract

Major depressive disorder (MDD) is a mental health disorder that can cause disability and functional impairment that standard-of-care (SOC) antidepressant therapies (ADTs) can take weeks to treat. Zuranolone is a neuroactive steroid and positive allosteric modulator of synaptic and extrasynaptic γ-aminobutyric acid (GABA) type A receptors approved as an oral, once-daily, 14-day treatment course in adults with postpartum depression and under investigation in adults with MDD. The phase 3 CORAL Study (NCT04476030) evaluated the efficacy and safety of zuranolone 50 mg co-initiated with SOC ADT (zuranolone+ADT) vs placebo co-initiated with SOC ADT (placebo+ADT) in adults with MDD. Patients were randomized 1:1 to once-daily, blinded zuranolone+ADT or placebo+ADT for 14 days, then continued open-label SOC ADT for 28 more days. The primary endpoint was change from baseline (CFB) in the 17-item Hamilton Rating Scale for Depression (HAMD-17) total score at Day 3. Among 425 patients in the full analysis set, CFB in HAMD-17 total score at Day 3 was significantly improved with zuranolone+ADT vs placebo+ADT (least squares mean [standard error], −8.9 [0.39] vs −7.0 [0.38]; *p* = 0.0004). The majority of patients receiving zuranolone+ADT that experienced treatment-emergent adverse events (TEAEs) reported mild or moderate events. The most common TEAEs present in ≥10% of patients in either zuranolone+ADT or placebo+ADT groups were somnolence, dizziness, headache, and nausea. These results demonstrate that zuranolone+ADT provided more rapid improvement in depressive symptoms compared with placebo+ADT in patients with MDD, with a safety profile consistent with previous studies. Clinical trial registration: ClinicalTrials.gov identifier: NCT04476030.

## Introduction

Major depressive disorder (MDD) is a mental health disorder associated with significant mortality, serious functional impairment, and reduced quality of life (QoL) [1, 2]. The global prevalence of MDD, often a recurrent condition, has increased worldwide in recent decades, more so during the COVID-19 pandemic [3–6]. Many patients with MDD do not receive treatment or are often undertreated. The estimated global prevalence of MDD is >193 million cases [3], and less than one-third of people living with depression in the United States (US) receive adequate treatment [7, 8].

Standard-of-care (SOC) antidepressant therapies (ADTs), such as selective serotonin reuptake inhibitors (SSRIs) and serotonin norepinephrine reuptake inhibitors (SNRIs), are efficacious but generally take weeks or months to improve depressive symptoms, which may lead to an increased risk of suicide and treatment discontinuation [9–11]. In the Sequenced Treatment Alternatives to Relieve Depression Study, mean time to remission in patients with MDD who received an SOC ADT ranged from 5.4 to 7.4 weeks [9]. Moreover, the potential for remission following acute treatment decreased and the rate of relapse increased with each subsequent acute ADT treatment step. Existing SOC ADTs also may not be suitable for some patients with MDD due to commonly reported adverse events (AEs), which may lead to non-adherence and decreased QoL [12–14]. Furthermore, although some rapid-acting ADTs like ketamine may produce rapid responses in patients with treatment-resistant depression [15], commonly reported AEs and abuse potential may limit their use [16]. Therefore, therapeutic options with novel mechanisms of action that can provide rapid responses with tolerable safety profiles to treat depressive symptoms are needed.

In the pursuit for novel treatments, different mechanisms of depression are being investigated, particularly disruption in the balance between excitatory glutamate and inhibitory γ-aminobutyric acid (GABA) signaling [17–22]. Several approved and investigational ADTs exhibit their effects on GABAergic/glutamatergic signaling, which are thought to contribute to the restoration of excitatory-inhibitory balance in the brain [21, 23, 24]. Neuroactive steroids (NASs) are hypothesized to rapidly restore network balance in brain areas dysregulated in depression by binding to GABA type A (GABA_A_) receptors (GABA_A_Rs). Brexanolone, a proprietary intravenous formulation of allopregnanolone, was the first NAS approved by the US Food and Drug Administration (FDA) for the treatment of postpartum depression (PPD) in patients aged 15 years or older [25]. Zuranolone (ZURZUVAE^TM^) is a positive allosteric modulator of synaptic and extrasynaptic GABA_A_Rs and the second NAS approved by the FDA as an oral, once-daily, 14-day treatment course in adults with PPD [26, 27] and under investigation (as a monotherapy or adjunct therapy) for adults with MDD in the LANDSCAPE clinical development program [27–31]. Notably, unlike brexanolone or allopregnanolone, zuranolone is a new chemical entity optimized for increased GABA_A_R selectivity, improved oral bioavailability, and a half-life consistent with once-daily dosing [27, 32].

As part of the LANDSCAPE program, the ongoing phase 3 SHORELINE Study (NCT03864614; publication of a manuscript describing interim data currently under review) was designed to assess the safety and tolerability of an open-label, 14-day treatment course of zuranolone 30 or 50 mg, as well as the need for repeat treatment courses for up to 1 year. Interim results from SHORELINE showed that most treatment-emergent AEs (TEAEs) were mild or moderate, and ~80% of patients who responded to the first course of zuranolone 50 mg and continued beyond Day (D)28 received a total of 1 or 2 treatment courses through ≤1 year of follow-up [33]. Other completed studies showed that zuranolone 50 mg vs placebo led to improvements in depressive symptoms observed at the earliest time point assessed (D3) in patients with MDD regardless of concomitant stable SOC ADT use [30, 31]. However, the efficacy and safety of zuranolone co-initiated with an SOC ADT were not investigated in the aforementioned studies.

Understanding the potential of zuranolone to rapidly improve depressive symptoms, including when co-initiated with an ADT, is important, because previous literature shows that individuals with MDD who achieve early treatment responses are likely to achieve better outcomes than those who do not achieve an early response or who have lingering or unresolved symptoms [34]. Co-initiating zuranolone with an SOC ADT may also result in improved efficacy, as NASs have a mechanism of action that is thought to be distinct from those of SOC ADTs [32, 35, 36]. The CORAL Study (NCT04476030) was designed to evaluate the potential rapid onset of effect and tolerability of zuranolone co-initiated with an open-label ADT (zuranolone+ADT) vs placebo co-initiated with an open-label ADT (placebo+ADT) administered once daily for 14 days in adults with MDD. Given the prevalent use of SSRI and SNRI ADTs, CORAL provides clinical context for the possibility of achieving a more rapid response by co-initiating zuranolone with an SOC ADT. CORAL also provides valuable additional safety data for zuranolone co-initiation with an ADT.

## Materials and methods

### Study design

The phase 3, randomized, double-blind, parallel-group, placebo-controlled CORAL Study evaluated the efficacy and safety of zuranolone+ADT in adults with MDD. The study period consisted of a screening period of ≤28 days, followed by a double-blind treatment course of 14 days (treatment period), and then a 28-day follow-up period with continued open-label SOC ADT treatment.

The study protocol and all amendments were approved by the appropriate Institutional Review Boards, and the study was performed in accordance with the ethical principles of the Declaration of Helsinki and guidelines of the International Council for Harmonisation of Technical Requirements for Pharmaceuticals for Human Use and Good Clinical Practice, as well as all applicable regulatory requirements. All patients provided written informed consent before beginning the study.

### Patients

During the screening period, the diagnosis of MDD was confirmed according to the Structured Clinical Interview for Diagnostic and Statistical Manual of Mental Disorders, Fifth Edition. Screening procedures included completion of the Massachusetts General Hospital Antidepressant Treatment Response Questionnaire and the 17-item Hamilton Rating Scale for Depression (HAMD-17). Eligible patients were 18–64 years old, were diagnosed with MDD with symptoms present for ≥4 weeks and had a HAMD-17 total score ≥24 at screening and D1 (prior to dosing). The full list of eligibility criteria is available in the Supplementary Material.

### Interventions

Each patient received prespecified, open-label ADTs, either an SSRI (sertraline, escitalopram, or citalopram) or an SNRI (duloxetine or desvenlafaxine) from D1 through the end of the study. Patients were stratified by the co-initiated ADT class (SSRI or SNRI) and randomized 1:1 to receive zuranolone 50 mg or matching placebo during the 14-day treatment period. The SOC ADT was assigned at the investigator’s discretion. The dose of ADT could be adjusted based on individual response at the discretion of the investigator.

Patients self-administered zuranolone or placebo orally once daily in the evening with fat-containing food for 14 days on an outpatient basis. At the discretion of the investigator, patients who did not tolerate zuranolone 50 mg could receive zuranolone 40 mg for the remainder of the treatment period; patients who did not tolerate zuranolone 40 mg were discontinued from receiving study drug. The dose reduction to 40 mg was prespecified per protocol, with dose reduction by 10 mg showing a positive benefit/risk profile.

### Endpoints

The primary endpoint was change from baseline (CFB) in the HAMD-17 total score at D3. The key secondary efficacy endpoint was CFB in HAMD-17 total score over the double-blind treatment period, using equal weights for the scheduled visits (D3, D8, D12, and D15).

Other secondary efficacy endpoints included CFB in HAMD-17 total score at D15 and D42; CFB in the HAMD-17 total score around the end of blinded treatment (using equal weights for the scheduled visits at D12, D15, and D18); HAMD-17 response, defined as ≥50% reduction from baseline in HAMD-17 total score at D15 and D42; time to first HAMD-17 response; and HAMD-17 remission, defined as HAMD-17 total score ≤7, at D15 and D42. Several other secondary endpoints were assessed and are described in the Supplementary Material.

### Assessments

HAMD-17 total score was measured at D1, D3, D8, D12, and D15 (during the double-blind treatment period) and at D18, D21, D28, D35, and D42 of the study (during the ADT continuation period). The effect size for the primary efficacy endpoint was calculated using Cohen’s d. Assessments related to patient-level clinical significance and the other secondary endpoints are described in the Supplementary Material.

Safety and tolerability were assessed by monitoring the incidence and severity of TEAEs as well as by monitoring vital signs, clinical laboratory measurements, and electrocardiograms. Suicidal ideation and behavior were evaluated using the Columbia-Suicide Severity Rating Scale (C-SSRS). Potential withdrawal symptoms following the discontinuation of study drug were monitored using the 20-item Physician Withdrawal Checklist (PWC-20).

### Statistical analysis

The safety set included all patients who received blinded study drug. The full analysis set (FAS) included all randomized patients in the safety set with a valid baseline and ≥1 postbaseline total score on at least one of the efficacy assessments (HAMD-17, Hamilton Rating Scale for Anxiety [HAM-A], Montgomery-Åsberg Depression Rating Scale [MADRS], or the 9-item Patient Health Questionnaire) or those with a valid baseline and ≥1 postbaseline value on the Clinical Global Impressions-Improvement score and/or Clinical Global Impression-Severity score.

A sample size of 382 evaluable patients was determined to provide 90% power to detect a statistically significant difference in the primary endpoint using a two-sided alpha level of 0.05, assuming the true difference is 3 points and a standard deviation (SD) of 9 points. Evaluable patients were those who were randomized and received blinded study drug and had a valid baseline and ≥1 postbaseline HAMD-17 assessment.

Continuous endpoints, including the primary and key secondary endpoints, were analyzed using a mixed effects model for repeated measures. Multiplicity adjustment of the key secondary endpoint was conducted by using the fixed sequence strategy [37]. Only if the primary endpoint was statistically significant at a two-sided 0.05 level could the key secondary endpoint be tested at the same level of significance. Analysis of the other secondary endpoints is detailed in the Supplementary Material. Efficacy endpoints, except for the primary and key secondary endpoint, were not adjusted for multiplicity, and differences for these endpoints were reported with nominal *p* values. All efficacy-related endpoints were analyzed using the FAS. All analyses and data outputs were generated using SAS^®^ (Cary, NC) version 9.4 or higher.

## Results

### Patient disposition, demographic, and baseline clinical characteristics

**Fig. 1:**
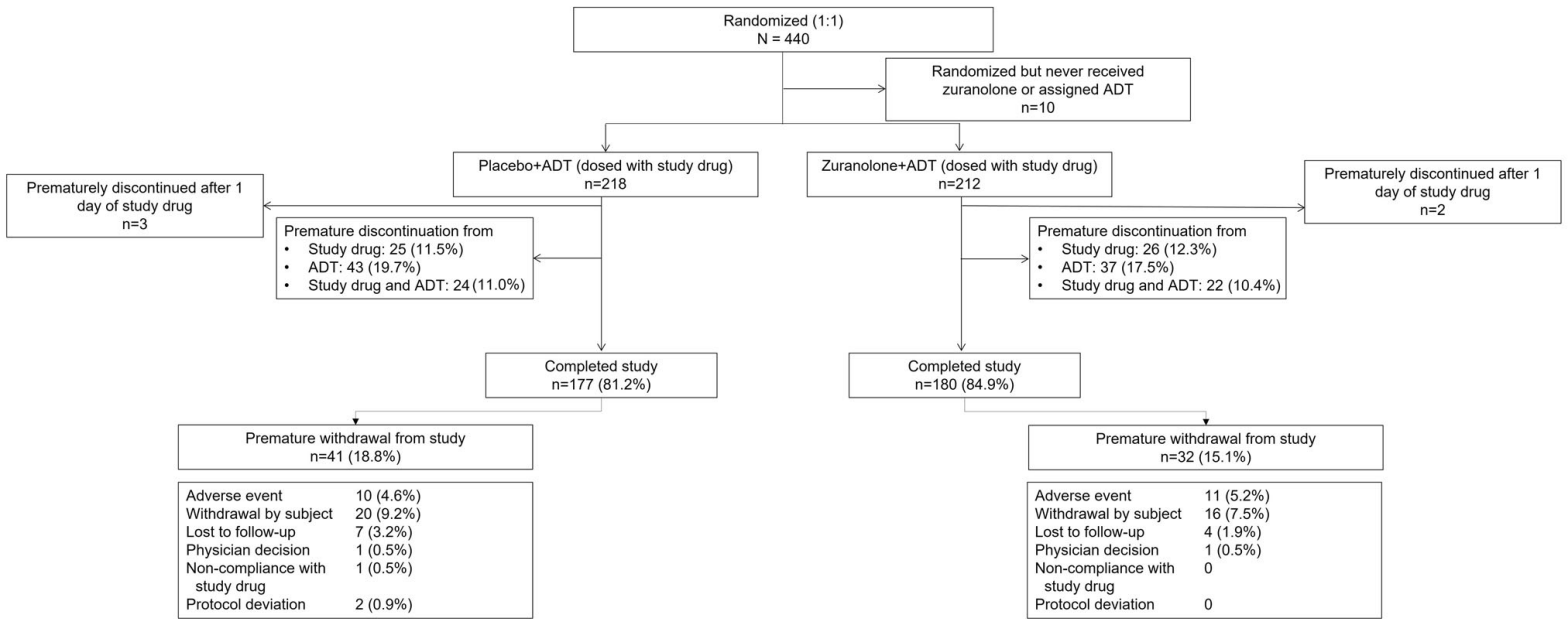
Patient disposition. ADT antidepressant therapy.

Of the 440 randomized patients, 430 received ≥1 dose of study drug (zuranolone+ADT, *n* = 212; placebo+ADT, *n* = 218; safety set) during the treatment period (Fig. 1); of these, five patients (*n* = 2 and *n* = 3, respectively) prematurely discontinued the study after D1, with no postbaseline efficacy data, leaving a total of 425 patients in the FAS (*n* = 210 and *n* = 215, respectively). A total of 186 (87.7%) patients who received zuranolone+ADT and 193 (88.5%) who received placebo+ADT completed treatment; 180 (84.9%) and 177 (81.2%) patients, respectively, completed the study.

Demographic and baseline clinical characteristics were comparable between the treatment groups (Table 1). Most patients in the zuranolone+ADT and placebo+ADT groups were female (60.8% and 64.2%, respectively), White (72.2% and 77.1%, respectively), and had a history of antidepressant use (54.2% and 55.0%, respectively). The mean age of patients who received zuranolone+ADT and placebo+ADT was 38.6 and 37.7 years, respectively. The median time since the start of the first episode in patients who received zuranolone+ADT and placebo+ADT was 4914 and 4838 days, respectively, and patients in both groups had experienced a median of 3 depressive episodes (including the current one). The mean (SD) HAMD-17 total score at baseline was 26.8 (2.5) in patients who received zuranolone+ADT and 26.6 (2.6) in patients who received placebo+ADT.

**Table 1:** Baseline demographics and patient characteristics (safety set).

Characteristic	Zuranolone+ADT *n* = 212	Placebo+ADT *n* = 218
Age, years, mean ± SD	38.6 ± 12.72	37.7 ± 12.28
Sex, *n* (%)		
Female	129 (60.8)	140 (64.2)
Male	83 (39.2)	78 (35.8)
Race, *n* (%)		
White	153 (72.2)	168 (77.1)
Black or African American	46 (21.7)	31 (14.2)
Asian	6 (2.8)	12 (5.5)
Other	3 (1.4)	3 (1.4)
>1 race	2 (0.9)	4 (1.8)
American Indian or Alaskan Native	1 (0.5)	0
Native Hawaiian or other Pacific Islander	1 (0.5)	0
Ethnicity, *n* (%)		
Not Hispanic or Latino	171 (80.7)	166 (76.1)
Hispanic or Latino	41 (19.3)	52 (23.9)
BMI, kg/m^2^, mean ± SD	29.1 ± 6.26	29.9 ± 6.44
HAMD-17 total score, mean ± SD	26.8 ± 2.51	26.6 ± 2.58
MADRS total score, mean ± SD	35.2 ± 4.70	34.9 ± 4.89
HAM-A total score, mean ± SD	19.9 ± 5.28	20.4 ± 5.80
CGI-S score, mean ± SD	5.0 ± 0.54	4.9 ± 0.57
Years since initial diagnosis of MDD, median (range)	6.95 (0.0–46.5)	7.10 (0.0–44.9)
History of any antidepressant use, *n* (%)		
Yes	115 (54.2)	120 (55.0)
No	97 (45.8)	98 (45.0)
Days since start of current episode, median (range)	282.0 (45–7817)	323.0 (11–4575)
Days since start of first episode, median (range)^a^	4914.0 (403–16990)	4838.0 (229–17794)
Number of depressive episodes experienced, median (range)^b^	3.0 (1–100)	3.0 (1–100)

*ADT* antidepressant therapy, *BMI* body mass index, *CGI-S* Clinical Global Impression-Severity, *HAM-A* Hamilton Rating Scale for Anxiety, *HAMD-17* 17-item Hamilton Rating Scale for Depression, *MADRS* Montgomery-Åsberg Depression Rating Scale, *MDD* major depressive disorder, *SD* standard deviation.

^a^Days since first depressive episode were calculated as days between the date of the first dose and the start date of the first depressive episode. ^b^Includes current episode.

In the safety set, a total of 210 patients who received zuranolone+ADT and 218 patients who received placebo+ADT received ≥1 dose of ADT. Patients who received zuranolone co-initiated SOC treatment were assigned ADTs in the following proportions: escitalopram (73/210; 34.8%), sertraline (66/210; 31.4%), desvenlafaxine (33/210; 15.7%), duloxetine (21/210; 10.0%), and citalopram (17/210; 8.1%); and SSRIs (156/210; 74.3%) and SNRIs (54/210; 25.7%). Patients who received placebo co-initiated SOC treatment were assigned ADTs in the following proportions: escitalopram (77/218; 35.3%), sertraline (53/218; 24.3%), desvenlafaxine (32/218; 14.7%), duloxetine (27/218; 12.4%), and citalopram (29/218; 13.3%); and SSRIs (159/218; 72.9%) and SNRIs (59/218; 27.1%).

### Primary endpoint

**Fig. 2:**
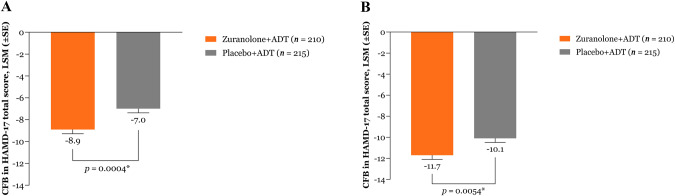
Change from baseline in HAMD-17 total score. Change from baseline in HAMD-17 total score at (**A**) Day 3 and (**B**) over the 14-day treatment period. Data are shown for the full analysis set using a mixed effects model for repeated measures. Change from baseline in HAMD-17 total score over the blinded treatment period was estimated using equal weights for the scheduled visits at Days 3, 8, 12, and 15. *p < 0.05. ADT antidepressant therapy, CFB change from baseline, HAMD-17 17-item Hamilton Rating Scale for Depression, LSM least squares mean, SE standard error.

Patients treated with zuranolone+ADT demonstrated a statistically significant improvement in depressive symptoms at D3 compared with those who received placebo+ADT, as assessed by CFB in HAMD-17 total score (least squares [LS] mean [standard error (SE)] CFB, −8.9 [0.39] vs −7.0 [0.38]; LS mean difference [SE] of −1.9 [0.55]; *p* = 0.0004; Cohen’s *d* = 0.38; Fig. 2A). These improvements at D3 were similar across subgroups defined by patient baseline characteristics (Fig. S1).

### Post hoc meaningful change threshold analysis

The patient-level clinical significance of CFB in HAMD-17 total score was determined by the meaningful change threshold (MCT), which was previously estimated to be −9.0 points for a similar patient population in the WATERFALL Study (NCT04442490; Supplementary Material) [38]. A greater proportion of patients treated with zuranolone+ADT vs placebo+ADT achieved a meaningful change from baseline (i.e., surpassed CFB in HAMD-17 of –9.0) at D3 (49.5% vs 37.1%; nominal *p* = 0.0108), and a numerically greater proportion achieved a meaningful change from baseline at D8 (62.9% vs 54.5%) and D15 (77.2% vs 72.6%; Fig. S2).

### Secondary endpoints

The key secondary endpoint (CFB in HAMD-17 total score over the blinded treatment period) demonstrated a statistically significant improvement in patients who received zuranolone+ADT compared with those who received placebo+ADT over the 14 days of treatment (LS mean [SE] CFB, −11.7 [0.40] vs −10.1 [0.39]; *p* = 0.0054; Fig. 2B).

**Fig. 3:**
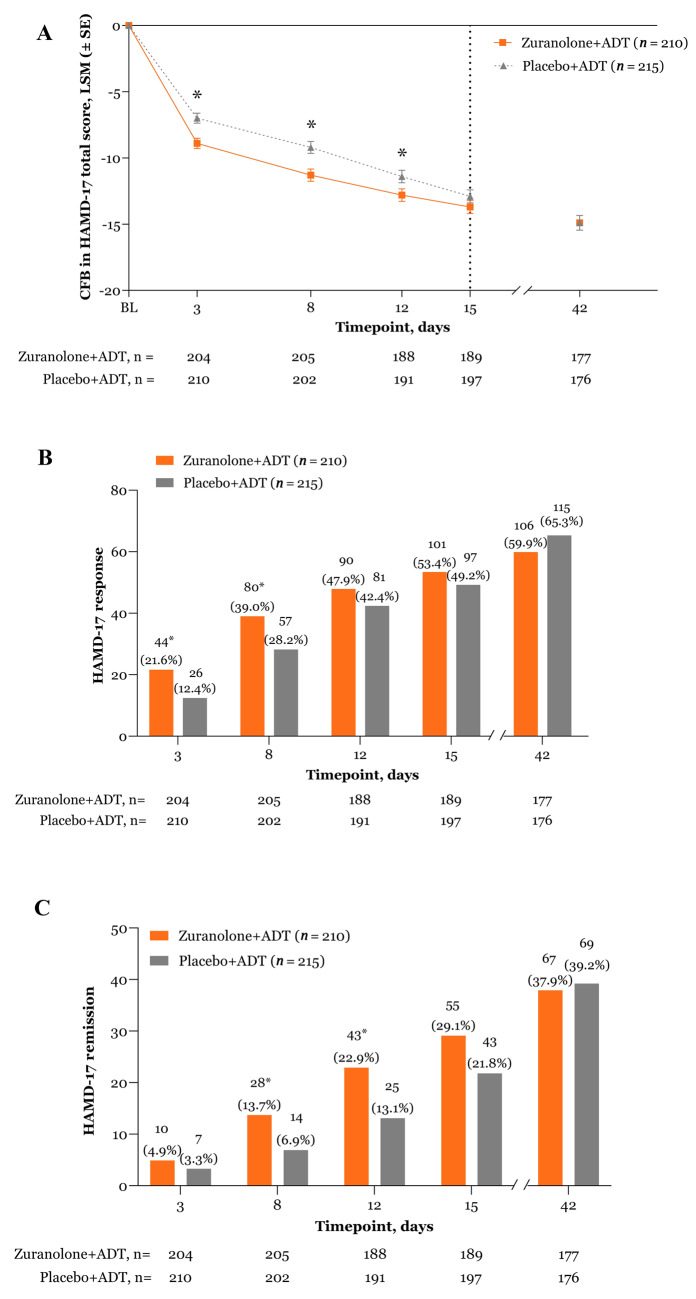
Improvement in depressive symptoms by time point and treatment group. Change from baseline in (**A**) HAMD-17 total score and (**B**) HAMD-17 response and (**C**) HAMD-17 remission. HAMD-17 response was defined as a ≥50% reduction from baseline in HAMD-17 total score. HAMD-17 remission was defined as HAMD-17 total score ≤7. Data are shown for the full analysis set using a mixed effects model for repeated measures. Day 3 is the primary endpoint; all other time points were not adjusted for multiplicity, and *p* values are considered nominal. The vertical dashed line signifies the end of the treatment period and the final assessment of the primary endpoint. *p < 0.05. ADT antidepressant therapy, CFB change from baseline, HAMD-17 17-item Hamilton Rating Scale for Depression, LSM least squares mean, SE standard error.

At D15, the LS mean (SE) CFB in HAMD-17 total score was −13.7 (0.50) in the zuranolone+ADT group and −12.9 (0.49) in the placebo+ADT group (LS mean difference [SE], −0.8 [0.70]; *p* = 0.2477). At D42, the LS mean (SE) reduction from baseline in HAMD-17 total score was −14.9 (0.56) for both groups (LS mean difference [SE], −0.1 [0.79]; *p* = 0.9248; Fig. 3A).

The percentages of patients who achieved HAMD-17 response in the zuranolone+ADT and placebo+ADT groups were 53.4% and 49.2%, respectively, at D15 (odds ratio [OR] [95% CI], 1.15 [0.78, 1.69]; *p* = 0.4946) and 59.9% and 65.3%, respectively, at D42 (OR [95% CI], 0.82 [0.55, 1.24]; *p* = 0.3579; Fig. 3B). A total of 158 (75.2%) patients in the zuranolone+ADT group and 157 (73.0%) patients in the placebo+ADT group achieved HAMD-17 response during the study, with estimated median time to first HAMD-17 response of 13 and 15 days, respectively.

In the zuranolone+ADT and placebo+ADT groups, respectively, the proportions of patients with HAMD-17 remission were 29.1% and 21.8% at D15 (OR [95% CI], 1.41 [0.89, 2.24]; *p* = 0.1417) and 37.9% and 39.2% at D42 (OR [95% CI], 0.94 [0.62, 1.44]; *p* = 0.7872; Fig. 3C). Ninety-eight (46.7%) patients who received zuranolone+ADT and 106 (49.3%) patients who received placebo+ADT achieved HAMD-17 remission during the study, with an estimated median time to first remission of 43 days in both treatment groups. Results for the other secondary endpoints are detailed in the Supplementary material, in Figs. S3, and S4, and in Table S1.

### Safety and tolerability

At least one TEAE was reported in 74.1% of patients who received zuranolone+ADT and in 65.6% of patients who received placebo+ADT (Table 2). Most TEAEs experienced by patients were mild or moderate, and no deaths occurred in the study. The most common TEAEs present in ≥10% of patients in the zuranolone+ADT or placebo+ADT groups were somnolence (18.4% vs 8.3%), dizziness (13.2% vs 7.3%), headache (11.8% vs 14.7%), and nausea (9.0% vs 23.4%). Two serious TEAEs were reported in this study, both in the zuranolone+ADT group (seizure like phenomenon at D7 that was considered related to blinded zuranolone by the investigator and exacerbation of chronic obstructive pulmonary disease at D23 that was considered not related to blinded zuranolone or the ADT [sertraline]; see Supplementary Material for more details).

**Table 2:** Summary of treatment-emergent adverse events^a^.

Safety parameters, *n* (%)	Zuranolone+ADT *n* = 212	Placebo+ADT *n* = 218
Any TEAE^b^	157 (74.1)	143 (65.6)
Mild	76 (35.8)	83 (38.1)
Moderate	73 (34.4)	55 (25.2)
Severe	8 (3.8)	5 (2.3)
Dose reduction due to TEAE	20 (9.4)^c^	6 (2.8)
Treatment discontinuation due to TEAE	14 (6.6)^d^	8 (3.7)
Study withdrawal due to TEAE	11 (5.2)	10 (4.6)
Serious TEAE	2 (0.9)^e^	0
Most common TEAEs (>5% in any group)		
Somnolence	39 (18.4)	18 (8.3)
Dizziness	28 (13.2)	16 (7.3)
Headache	25 (11.8)	32 (14.7)
Insomnia	21 (9.9)	17 (7.8)
Dry mouth	20 (9.4)	19 (8.7)
Nausea	19 (9.0)	51 (23.4)
Fatigue	18 (8.5)	11 (5.0)
Diarrhea	13 (6.1)	21 (9.6)
Decreased appetite	12 (5.7)	7 (3.2)
Sedation	12 (5.7)	6 (2.8)
Tremor	11 (5.2)	3 (1.4)

*ADT* antidepressant therapy, *TEAE* treatment-emergent adverse event.

^a^TEAEs were reported for the safety set. The safety set was defined as all randomized patients administered blinded zuranolone 50 mg or placebo. TEAEs were ordered by descending incidence in the zuranolone+ADT group.

^b^Maximum severity of the TEAE.

^c^The most common TEAEs (>1 patient) leading to dose reduction in the zuranolone+ADT group were somnolence, dizziness, tremor, fatigue, and nausea.

^d^The most common TEAEs (>1 patient) leading to treatment discontinuation in the zuranolone+ADT group were sedation, dizziness, somnolence, and nausea.

^e^One patient experienced a seizure-like phenomenon ~1 h after receipt of zuranolone 50 mg on Day 7; the event was assessed as related to zuranolone. Another patient experienced exacerbation of their chronic obstructive pulmonary disease on Day 23 of the ADT continuation period; the event was assessed as not related to zuranolone or ADT and resolved 2 days later.

No increases from baseline in suicidal ideation/behavior signals, as assessed by the C-SSRS, or withdrawal symptoms, as assessed by the PWC-20, were identified in patients who received zuranolone+ADT. The proportion of patients who had suicidal ideation at any postbaseline visit from D3 through D42 ranged from 4.8% to 11.7% in the zuranolone+ADT group and from 6.0% to 12.4% in the placebo+ADT group (Table S2). No patients who received placebo+ADT experienced suicidal behavior. For patients who received zuranolone+ADT, one experienced suicidal behavior at baseline and a different patient experienced suicidal behavior at D28. Mean decreases from baseline in PWC-20 total score were observed at D18 (−0.6 for zuranolone+ADT patients, −1.1 for placebo+ADT patients), D21 (−0.5, −1.7), and D28 (−0.8, −1.0) and were similar between the treatment groups (Table S3).

## Discussion

The CORAL Study was designed to assess the rapid improvement in depressive symptoms through co-initiation of zuranolone with an SOC ADT. The primary endpoint of CFB in HAMD-17 total score at D3 demonstrated significantly greater improvement in depressive symptoms with zuranolone+ADT vs placebo+ADT, providing clinical evidence for zuranolone as a potential rapid-acting treatment for patients with MDD who are initiating SOC ADTs. The effect size for the primary endpoint at D3 was 0.38. While not directly comparable, the reported mean effect size of varying primary endpoints for 34 ADT registration trials conducted after 2000 was 0.29 [39]. The results from CORAL are consistent with previous studies in which patients who were administered zuranolone as a monotherapy or as an adjunct therapy to stable ADTs experienced significant improvements in depressive symptoms at the primary endpoint, and the majority of TEAEs were mild or moderate in severity [29–31, 33, 40].

The key secondary endpoint examined this temporal benefit with zuranolone+ADT more broadly, showing these improvements to be statistically significant over the full blinded treatment period, and not limited only to D3. Other secondary endpoints that were not nominally significant or part of the formal testing procedure showed that 53.4% of patients who received zuranolone+ADT achieved HAMD-17 response vs 49.2% of those receiving placebo+ADT at D15, with a median time to first response of 13 and 15 days, respectively; in addition, 29.1% and 21.8% of patients in the zuranolone+ADT and placebo+ADT groups, respectively, achieved HAMD-17 remission at D15. Most of the other secondary endpoints were not nominally significant.

The rapid improvement in depressive symptoms experienced by patients receiving zuranolone+ADT was also clinically meaningful, which was quantified using the MCT. The MCT assesses patient-level clinical significance based on patients achieving a threshold CFB in HAMD-17, which was estimated for a similar patient population in the WATERFALL Study to be –9.0 [38]. A greater proportion of patients treated with zuranolone+ADT vs placebo+ADT achieved a meaningful CFB in HAMD-17 total score (i.e., surpassed the MCT of –9.0) at D3 (49.5% vs 37.1%; nominal *p* = 0.0108), and a numerically greater proportion achieved a meaningful change from baseline at D8 (62.9% vs 54.5%) and D15 (77.2% vs 72.6%). Previous reports investigating the role of ADTs in patients with MDD empirically determined that a clinically significant MCT for the HAMD-17 total score ranged from –5.0 to –8.0 [41–47]. MCT values generally vary across studies, in part likely due to variable baseline depression severities between patient populations [44]. Prior findings revealed that baseline depression severity positively correlated to greater improvement in CFB HAMD-17 total score [41, 44], suggesting that an MCT value derived from populations with comparable baseline depression severities would be an appropriate benchmark for assessing the CORAL Study. Much like that of CORAL, the patient population of the WATERFALL Study had severe depression at baseline (mean baseline HAMD-17 total scores of 26.8 and 26.9 in the zuranolone and placebo groups, respectively) and was therefore used to assess the clinical significance of CFB HAMD-17 total score in the present study. Of note, a threshold-based measurement such as MCT does not offer insights into patients who marginally failed to achieve a –9.0 CFB in HAMD-17 total score. Patients just below or just above this threshold may have experienced similar improvements in depressive symptoms. Together, these data suggest that zuranolone as a co-initiation therapy to SOC ADT compared with ADT monotherapy led to rapid and clinically significant response at D3, which was maintained over the 14-day treatment course [31, 38].

Patients administered zuranolone vs placebo, each co-initiated with an SOC ADT, experienced mild to moderate AEs consistent with the known safety profiles of the study drugs [40, 48]. The most common TEAEs (present in ≥10% of patients in either zuranolone+ADT or placebo+ADT groups) were somnolence, dizziness, headache, and nausea. Notably, a lower incidence of gastrointestinal AEs (e.g., nausea, diarrhea) was observed with zuranolone+ADT vs placebo+ADT. No increases from baseline in suicidal ideation or behavior and withdrawal symptoms (as assessed by C-SSRS and PWC-20, respectively) were reported throughout the study.

A limitation of the CORAL Study is that these results may not be generalizable to all patients with MDD, as adults with treatment-resistant depression or those at significant risk of suicide (or attempted suicide within the current episode) were not enrolled. Another limitation of CORAL and other zuranolone studies is that a patient’s AE profile may have had an unblinding effect of revealing treatment with zuranolone+ADT. The large placebo response commonly observed in depression studies was also observed in CORAL [49–51]. The relatively high frequency of study visits in the CORAL Study (5 visits during the first 15 days) may have contributed to the placebo response observed [52], consistent with meta-analyses showing that twice-weekly psychosocial interventions are more effective than once-weekly interventions in treating depression [53, 54]. Clinical visits in the real world are often less frequent than in clinical studies; a meta-analysis found that more frequent follow-up assessments in placebo-controlled ADT trials were associated with a large placebo response [52]. Additionally, a systematic literature review revealed that recent MDD trials in the US had a visit frequency of approximately 0.8 visits per week, which is considerably lower than the rate (~2.3 visits per week) in the CORAL Study [55]. In addition, co-initiation of ADTs at baseline in both groups may have created some expectation bias due to the knowledge that all patients were receiving active treatment—potentially further contributing to a placebo response. Despite these study limitations and the large placebo response, co-initiation of zuranolone with an SOC ADT demonstrated a more rapid treatment response over existing oral ADTs alone. This may be particularly useful to patients for whom a chronically administered ADT may be appropriate and a rapid improvement is clinically indicated.

Given the considerable heterogeneity of MDD [56, 57], one goal of the LANDSCAPE clinical development program has been to assess the utility of zuranolone in multiple clinical scenarios. Overall, the majority of patients receiving zuranolone+ADT experienced mild to moderate TEAEs, presenting a safety profile consistent with other studies in the program. When co-initiating with an SOC antidepressant—mimicking common real-world practice patterns—patients treated with zuranolone demonstrated rapid improvement in depressive symptoms.

### Supplementary information

Supplemental Material
