# Supplementary material for: Efficacy and safety of zuranolone co-initiated with an antidepressant in adults with major depressive disorder: results from the phase 3 CORAL study
Source: Neuropsychopharmacology. 2023 Oct 24;49(2):467–75. doi: 10.1038/s41386-023-01751-9 (PMC10724299; doi:10.1038/s41386-023-01751-9)
Supplement: Supplementary file 1 — Supplemental Material [file 41386_2023_1751_MOESM1_ESM.pdf]

Supplemental Material

Efficacy and safety of zuranolone co-initiated with an antidepressant in adults with major depressive disorder: results from the phase 3 CORAL Study

Sagar V. Parikh, MD[1]; Scott T. Aaronson, MD[2]; Sanjay J. Mathew, MD[3]; Gustavo Alva, MD[4]; Charles DeBattista, MD[5]; Stephen Kanes, MD, PhD[6]; Robert Lasser, MD[7]; Amy Bullock, PhD[7]; Mona Kotecha, MD[8]; JungAh Jung, PhD[7]; Fiona Forrestal, MSc[8]; Jeff Jonas, MD[7]; Theresa Vera, PhD[7]; Bridgette Leclair, PharmD[8]; James Doherty, PhD[7]

[1]Department of Psychiatry, University of Michigan, Ann Arbor, MI; [2]Institute for Advanced Diagnostics and Therapeutics, Sheppard Pratt, Baltimore, MD; [3]Menninger Department of Psychiatry and Behavioral Sciences, Baylor College of Medicine, Houston, TX; [4]ATP Clinical Research, Costa Mesa, CA; [5]General Psychiatry and Psychology, Stanford University School of Medicine, Stanford, CA; [6]EmbarkNeuro, Oakland, CA; [7]Sage Therapeutics, Inc., Cambridge, MA; [8]Biogen, Cambridge, MA

Address correspondence to:
Sagar V. Parikh, MD, FRCPC
Department of Psychiatry, University of Michigan
Rachel Upjohn Bldg, Rm 1303
4250 Plymouth Road
Ann Arbor, MI 48109-2700
Phone: (734) 232-0175
Email: parikhsa@umich.edu

TABLE OF CONTENTS

Inclusion criteria 3

Exclusion criteria 4

Other secondary endpoints 7

Serious treatment-emergent adverse events 8

Table S1 Summary of other secondary efficacy endpoints 11

Table S2 Proportion of patients with suicidal ideation or behavior at each C-SSRS assessment time point 12

Table S3 Change from baseline in the PWC-20 total score through Day 28 13

Fig. S1 Forest plot of treatment differences in HAMD-17 total score at Day 3 (full analysis set) 14

Fig. S2 Clinically significant change from baseline in HAMD-17 total score by time point and treatment group 15

Fig. S3 Change from baseline in MADRS total score 16

Fig. S4 CGI-I response by study visit 17

Inclusion criteria

- Patient signed an informed consent form prior to any study-specific procedures being performed.
- Patient was male or female, 18–64 years of age.
- Patient was in good physical health and had no clinically significant findings, as determined by the investigator on physical examination, 12-lead electrocardiogram (ECG), or clinical laboratory tests.
- Patient agreed to adhere to the study requirements.
- Patient had a diagnosis of major depressive disorder (MDD), as diagnosed by Structured Clinical Interview for Diagnostic and Statistical Manual of Mental Disorders, Fifth Edition (DSM-5) Clinical Trial Version, with symptoms that had been present for at least a 4-week period.
- Patient had a 17-item Hamilton Rating Scale for Depression (HAMD-17) total score ≥24 at screening and Day 1 (prior to dosing).
- Patient was willing to delay start of any antidepressant therapy (ADT; except per protocol), anxiolytic, anti-insomnia, psychostimulant, prescription opioid regimen, or new psychotherapy (including Cognitive Behavioral Therapy for Insomnia) until after study completion. Patients receiving psychotherapy had received therapy on a regular schedule for at least 60 days prior to Day 1 and intended to maintain that schedule for the duration of the study.
- Female patient agreed to use one of the following methods of highly effective contraception during participation in the study and for 30 days following the last dose of investigational product (IP) unless she was postmenopausal (defined as at least 12 months of spontaneous amenorrhea without an alternative medical cause, confirmed by follicle-stimulating hormone >40 mIU/mL) and/or surgically sterile (bilateral oophorectomy, hysterectomy, and/or bilateral salpingectomy) or did not engage in sexual relations that carried a risk of pregnancy (did not include abstinence).
    - Combined (estrogen- and progestogen-containing) oral, intravaginal, or transdermal hormonal contraception associated with inhibition of ovulation.
    - Oral, injectable, or implantable progestogen-only hormonal contraception associated with inhibition of ovulation.
    - Intrauterine device.
    - Intrauterine hormone-releasing system.

    - Laparoscopic or abdominal bilateral tubal occlusion procedure (including bilateral tubal ligation).
    - Hysteroscopic bilateral tubal occlusion procedure performed at least 3 months prior to screening.
    - Vasectomized partner (performed at least 3 months prior to screening).
- Female patient who was breastfeeding at screening or on Day 1 (prior to administration of IP) was willing to temporarily cease giving breastmilk to her child(ren) from just prior to receiving IP on Day 1 until 7 days after the last dose of IP.
- Male patient agreed to use an acceptable method of effective contraception for the duration of the study and for 5 days after receiving IP, unless he did not engage in sexual relations that carried a risk of pregnancy. Acceptable methods of effective contraception for male patients included history of vasectomy (performed at least 3 months prior to screening) or a condom with spermicide used together with highly effective female contraceptive methods if the female partner was of childbearing potential (see earlier criteria for acceptable contraception methods).
- Male patient was willing to abstain from sperm donation during the treatment period and for 5 days after receiving the last dose of IP.
- Patient agreed to refrain from using drugs of abuse and alcohol for the duration of the study.
- Patient was willing, able, and eligible to take at least 1 of the 5 specified ADTs (an eligible ADT was an ADT that had not been taken during the current depressive episode and for which the patient had no contraindications; further, a patient was not eligible for citalopram if escitalopram had been taken during the current depressive episode, and vice versa).

Exclusion criteria

- Patient was currently at significant risk of suicide, as judged by the investigator, or had attempted suicide associated with the current episode of MDD.
- Patient had onset of the current depressive episode during pregnancy or 4 weeks postpartum, or the patient presented for screening during the 6-month postpartum period.
- Patient had a recent history or active clinically significant manifestations of metabolic; hepatic; renal; hematologic; pulmonary; cardiovascular; gastrointestinal; musculoskeletal; dermatological; urogenital; neurological; or eyes, ears, nose, and throat

disorders; or any other acute or chronic condition that, in the investigator's opinion, would have limited the patient's ability to complete or participate in this clinical study. A body mass index (BMI) ≤18 or ≥45 kg/m^2 at screening was exclusionary; a BMI of 40–44.9 kg/m^2, inclusive, at screening was subject to a broader evaluation of medical comorbidities as described above.

- Patient had treatment-resistant depression, defined as persistent depressive symptoms despite treatment with adequate doses of ADT within the current major depressive episode (excluding antipsychotics) from 2 different classes for at least 4 weeks of treatment. The Massachusetts General Hospital Antidepressant Treatment Response Questionnaire was used for this purpose.
- Patient had vagus nerve stimulation or electroconvulsive therapy or had taken ketamine within the current major depressive episode.
- Patient was receiving Cognitive Behavioral Therapy for Insomnia within 28 days prior to Day 1.
- Patient had a known allergy to zuranolone, allopregnanolone, or related compounds.
- Patient had taken ADT within 30 days prior to Day 1, and/or had taken fluoxetine within 60 days prior to Day 1.
- Female patient had a positive pregnancy test or confirmed pregnancy.
- Patient had a clinically significant abnormal 12-lead ECG at the screening or baseline visit. Mean QT corrected according to Fridericia's formula (QTcF) of >450 msec in male patient or >470 msec in female patient.
- Patient had active psychosis per investigator assessment.
- Patient had a medical history of seizures.
- Patient had a medical history of bipolar disorder, schizophrenia, and/or schizoaffective disorder.
- Patient had a history of severe substance use disorder (including of benzodiazepines) diagnosed using DSM-5 criteria in the 12 months prior to screening, or patient had a history of mild or moderate substance use disorder not in sustained remission for at least 6 months prior to screening.
- Patient had exposure to another investigational medication or device within 30 days prior to screening.
- Patient had previously received brexanolone or participated in a zuranolone or brexanolone clinical trial.

- Patient had used any known strong inhibitors of cytochrome P450 (CYP3A4) within 28 days or 5 half-lives (whichever was longer) or consumed grapefruit juice, grapefruit, Seville oranges, or products containing these within 14 days prior to Day 1.
- Patient had used any strong CYP3A inducer, such as rifampin, carbamazepine, enzalutamide, mitotane, phenytoin, or St. John's Wort, within 28 days prior to Day 1.
- Patient had a positive drug and/or alcohol screen at screening or on Day 1 prior to dosing.
- Patient planned to undergo elective surgery before completion of the Day 42 visit.
- Patient was taking benzodiazepines, barbiturates, or gamma-aminobutyric acid (GABA) type A modulators (eg, eszopiclone, zopiclone, zaleplon, and zolpidem) within 28 days prior to Day 1 or had been using these agents daily or near daily (≥4 times per week) for more than 1 year. Patient was taking any benzodiazepine or GABA modulator with a half-life ≥48 hours (eg, diazepam) from 60 days prior to Day 1.
- Patient was taking non-GABA anti-insomnia medications (eg, prescribed therapeutics specifically for insomnia and/or over-the-counter sleep aids) or first-generation or second-generation (typical/atypical) antipsychotics within 14 days prior to Day 1. Note that non-sedating antihistamines were permitted.
- Patient had been diagnosed with and/or treated for any type of cancer (excluding basal cell carcinoma and melanoma *in situ*) within the year prior to screening.
- Patient had a history of sleep apnea.
- Patient had gastric bypass surgery, a gastric sleeve or lap band, or any related procedures that interfere with gastrointestinal transit.
- Patient was taking psychostimulants (eg, methylphenidate, amphetamine) or opioids, regularly or as needed, within 28 days prior to Day 1.
- Patient was a dependent of the sponsor, investigator, investigator's deputy, or study site staff.
- Patient was expected to perform night shift work during the 14-day treatment period.
- Patient had detectable hepatitis B surface antigen, anti–hepatitis C virus and positive hepatitis C virus viral load, or human immunodeficiency virus antibody at screening.

Patient-level clinical significance

Patient-level clinical significance of change from baseline (CFB) in HAMD-17 total score was determined by applying the meaningful change threshold (MCT) at Day (D) 3, D8, and D15. The MCT was previously calculated for a similar patient population in the WATERFALL Study

(NCT04442490)[1,2] based on the weighted mean CFB data observed among those reporting 1-unit improvement in Clinical Global Impression-Severity (CGI-S; rounded to an integer, as an MCT needs to be achievable at the patient level). The estimated MCT reflects a meaningful CFB that is substantially better than the CFB observed among patients reporting no change in CGI-S (ie, no overlap in 95% CI across no change and 1-unit change categories in zuranolone+ADT and placebo+ADT groups combined)[2].

Randomization, blinding, and patient monitoring for drug adherence

Randomization was performed centrally using an Interactive Response Technology System. Randomization schedules were generated by an independent statistician and kept strictly confidential, accessible only to authorized personnel until the time of unblinding.

Self-administration of the blinded study drug was monitored by a medication adherence monitoring platform on smartphones to visually confirm the study drug ingestion. Patients received a reminder within a predefined time window to take the study drug while using the application. Patients followed a series of prescribed steps to visually confirm their ingestion of the study drug. The application recorded the date and time of administration and any missed doses.

Other secondary endpoints

Other secondary efficacy endpoints, assessed using the full analysis set, included CFB in Montgomery-Åsberg Depression Rating Scale (MADRS) total score at Day 15; MADRS response, defined as ≥50% reduction from baseline in MADRS total score, at Day 15; MADRS remission, defined as MADRS total score ≤10, at Day 15; Clinical Global Impression-Improvement (CGI-I) response of “very much improved” or “much improved” from baseline at Days 3 and 15; CFB in Clinical Global Impression-Severity (CGI-S) at Day 15; CFB in Hamilton Rating Scale for Anxiety (HAM-A) total score at Day 15; and CFB in depressive symptoms at Day 15 as assessed by the 9-item Patient Health Questionnaire (PHQ-9).

CGI-S was assessed at Days 1, 3, 8, 12, 15, 21, 28, 35, and 42. MADRS and HAM-A were assessed at Days 1, 8, 15, 28, and 42. CGI-I was evaluated at Days 3, 8, 12, 15, 21, 28, 35, and 42. PHQ-9 was evaluated at Days 1, 3, 8, 15, 28, and 42.

HAMD-17 response, HAMD-17 remission, MADRS response, MADRS remission, and CGI-I response were analyzed using generalized estimating equation methods. The Kaplan-Meier method was employed to estimate time to first HAMD-17 response, with the number and percentage of patients who had a response or who were censored included in the analysis.

Similarly the Kaplan-Meier method was employed to estimate the time to first HAMD-17 remission.

The CFB in MADRS total score is shown in **Fig. S2**. At Day 8, the least squares (LS) mean (standard error [SE]) CFB in MADRS total score showed nominally significant improvement for zuranolone+ADT (−13.6 [0.71]) vs placebo+ADT patients (−11.2 [0.71]; LS mean difference [SE], −2.4 [1.00]; p=0.0167). At Day 15, the LS mean (SE) CFB in MADRS total score was −17.2 (0.76) for patients receiving zuranolone+ADT and −15.9 (0.75) for those receiving placebo+ADT (LS mean difference [SE], −1.3 [1.06]; p=0.2322). At Day 15, the proportion of patients who achieved MADRS response was 51.6% in the zuranolone+ADT group and 48.2% in the placebo+ADT group (odds ratio [95% CI], 1.13 [0.76, 1.68]; p=0.5439). The proportion of patients who achieved MADRS remission at Day 15 was 30.9% in the zuranolone+ADT group and 28.4% in the placebo+ADT group (odds ratio [95% CI], 1.09 [0.70, 1.69]; p=0.7054).

At Day 3, the proportion of patients achieving a CGI-I response was 22.9% in the zuranolone+ADT group and 12.9% in the placebo+ADT group and were nominally significantly different between groups (odds ratio [95% CI], 2.05 [1.21, 3.47]; p=0.0079; **Fig. S3**). At Day 15, >50% of patients in both treatment groups achieved a CGI-I response (zuranolone+ADT, 56.6%; placebo+ADT, 54.3%; odds ratio [95% CI], 1.09 [0.74, 1.62]; p=0.6588).

The LS mean (SE) CFB in HAMD-17 total score around end of blinded treatment (over Days 12, 15, and 18) were −13.2 (0.46) and −12.7 (0.45) in patients who received zuranolone+ADT and placebo+ADT, respectively (p=0.4458). The LS mean difference [SE] in PHQ-9 Total Score at Day 15 was −8.9 (0.44) and −8.7 (0.44) in patients who received zuranolone+ADT and placebo+ADT, respectively (p=0.7758). These additional secondary endpoints are also summarized in **Table S1.**

Serious treatment-emergent adverse events

Two (0.9%) patients in the zuranolone+ADT group experienced a serious treatment-emergent adverse event. Both participants were assigned to a selective serotonin reuptake inhibitor.

One patient (age 49, White male) experienced an event of seizure like phenomena approximately 1 hour after receiving zuranolone 50 mg on Day 7. The patient initiated treatment with zuranolone and escitalopram on Day 1. At the time of the event, the patient experienced dissociation and disorientation. There was no loss of consciousness and no bowel or bladder incontinence during or after the episode. This event resolved after approximately 22 minutes,

and the patient left the emergency room without evaluation. Zuranolone was discontinued due to the event on the same day. The patient continued escitalopram 10 mg and completed the study. The investigator considered this seizure like phenomena not related to escitalopram as the symptoms subsided with continued use of it. Notably, the patient had no history of seizures or other medical history that could have possibly contributed to the event, and the patient did not miss any scheduled doses of zuranolone (the patient received 7 doses, once daily, by the time the event onset occurred on Day 7). Based on review of available data and given the temporal relationship between zuranolone dosing and event onset, a causal association of zuranolone to the seizure like phenomena cannot be completely ruled out; however, a definitive causal association is confounded by concomitant treatment with escitalopram, which is associated with events such as seizures, confusion, disorientation, delirium, dissociation, amnesia, muscle weakness, and malaise. To further understand the event, an external expert epileptologist was also consulted, who opined that the event was that of a confusional state at peak drug effect, instead of seizure.

The other patient (age 57, White female) experienced exacerbation of chronic obstructive pulmonary disease on Day 23 of sertraline 100 mg dosing during the open-label ADT Continuation Period. The event resolved on Day 25 and was assessed as not related to either blinded zuranolone administration or the assigned ADT by the investigator.

References

1 Clayton AH, Lasser R, Parikh SV, Iosifescu DV, Jung J, Kotecha M, et al. Zuranolone for the treatment of adults with major depressive disorder: a randomized, placebo-controlled phase 3 trial. Am J Psychiatry. 2023;180(9):676-84.

2 Huang M-Y, Acaster S, Fridman M, Suthoff E, Chen S-Y, Kornstein S. Zuranolone in major depressive disorder (MDD): minimal important difference (MID) and meaningful change threshold (MCT) on the 17-item Hamilton Rating Scale for Depression (HAMD-17). Psych Congress; 17–20 September 2022; New Orleans, LA, USA. 2022;120.

Table S1 Summary of other secondary efficacy endpoints

	Zuranolone+ADT (n=210)		Placebo+ADT (n=210)			
**Secondary endpoint**	**n**	**LS mean CFB (SE)**	**n**	**LS mean CFB (SE)**	**Absolute change/difference**	**p-value**
CFB in CGI-S score at Day 15	189	−1.9 (0.09)	197	−1.7 (0.09)	−0.2	0.1993
CFB in HAM-A total score at Day 15	188	−9.5 (0.44)	197	−9.0 (0.44)	−0.5	0.4188
CFB in MADRS total score at Day 15	188	−17.2 (0.76)	197	−15.9 (0.75)	−1.3	0.2322
CFB in PHQ-9 total score at Day 15	188	−8.9 (0.44)	196	−8.7 (0.44)	−0.2	0.7758
	**n/N**	**%**	**n/N**	**%**	**OR (95% CI)**	**p-value**
HAMD-17 response at Day 12	90/188	47.9	81/191	42.4	1.22 (0.82, 1.82)	0.3244
HAMD-17 response at Day 15	101/189	53.4	97/197	49.2	1.15 (0.78, 1.69)	0.4946
HAMD-17 response at Day 18	96/181	53.0	109/190	57.4	0.87 (0.58, 1.29)	0.4783
HAMD-17 response at Day 42	106/177	59.9	115/176	65.3	0.82 (0.55, 1.24)	0.3579
HAMD-17 remission at Day 15	55/189	29.1	43/197	21.8	1.41 (0.89, 2.24)	0.1417
HAMD-17 remission at Day 42	67/177	37.9	69/176	39.2	0.94 (0.62, 1.44)	0.7872

Other secondary efficacy endpoints were not adjusted for multiplicity, and hence all p-values are considered nominal. HAMD-17 response was defined as CFB (improvement) in HAMD-17 total score ≥50%. HAMD-17 remission was defined as HAMD-17 total score ≤7. CGI-I response was defined as “much” or “very much” improved from baseline.

ADT antidepressant therapy, CFB change from baseline, CGI-I Clinical Global Impression-Improvement, CGI-S Clinical Global Impression-Severity, CI confidence interval, HAM-A Hamilton Rating Scale for Anxiety, HAMD-17 17-item Hamilton Rating Scale for Depression, LS least squares, MADRS Montgomery-Åsberg Depression Rating Scale, OR odds ratio, PHQ-9 9-item Patient Health Questionnaire, SE standard error.

Table S2 Proportion of patients with suicidal ideation or behavior at each C-SSRS assessment time point

	Zuranolone+ADT N=212			Placebo+ADT N=218		
**Study visit day**	**n**	**Suicidal ideation**	**Suicidal behavior**	**n**	**Suicidal ideation**	**Suicidal behavior**
Baseline[a]	212	104 (49.1)	1 (0.5)	218	98 (45.0)	0
3	205	19 (9.3)	0	209	26 (12.4)	0
8	205	24 (11.7)	0	203	23 (11.3)	0
12	188	17 (9.0)	0	193	13 (6.7)	0
15	189	14 (7.4)	0	198	14 (7.1)	0
18	182	17 (9.3)	0	190	12 (6.3)	0
21	166	18 (10.8)	0	173	16 (9.2)	0
28	180	15 (8.3)	1 (0.6)	184	11 (6.0)	0
35	167	8 (4.8)	0	172	14 (8.1)	0
42	177	11 (6.2)	0	176	16 (9.1)	0

Data shown as n (%). [a]Baseline considers all assessments prior to the first dose of blinded investigational product, excluding lifetime assessment, and the worst value for each question is counted.

ADT antidepressant therapy, C-SSRS Columbia-Suicide Severity Rating Scale.

Table S3 Change from baseline in the PWC-20 total score through Day 28

	Zuranolone+ADT n=212		Placebo+ADT n=218	
**Study visit**	**Observed**	**CFB[a]**	**Observed**	**CFB[a]**
Day 1	13.7 ± 8.20	N/A	14.8 ± 8.53	N/A
Day 15	7.0 ± 6.16	N/A	7.3 ± 6.03	N/A
Day 18	6.5 ± 5.80	−0.6 ± 4.31	6.3 ± 5.81	−1.1 ± 3.76
Day 21	6.5 ± 5.91	−0.5 ± 5.25	6.1 ± 5.52	−1.7 ± 4.48
Day 28	6.5 ± 6.20	−0.8 ± 5.91	6.7 ± 5.55	−1.0 ± 5.12

Data shown as mean ± SD. [a]Baseline refers to the first assessment after last dose within 1 day past last dose of blinded treatment. Higher or increased PWC-20 total scores are suggestive of more severe withdrawal symptoms.

ADT antidepressant therapy, CFB change from baseline, N/A not applicable, PWC-20 20-item Physician Withdrawal Checklist, SD standard deviation.

	n	LSM difference (SE)	95% CI
**HAMD-17 total score**	414	−1.9 (0.6)	−3.0, −0.9
**Age**			
18–24 years	52	−3.6 (1.5)	−6.6, −0.7
25–50 years	276	−1.9 (0.7)	−3.2, −0.6
51–64 years	85	−1.5 (1.3)	−4.0, 1.0
**Sex**			
Male	155	−1.9 (0.9)	−3.7, −0.1
Female	258	−1.9 (0.7)	−3.2, −0.5
**Race**			
White	309	−1.9 (0.6)	−3.1, −0.7
Black or African American	74	−2.7 (1.6)	−5.9, 0.6
Other	30	−1.3 (1.9)	−5.3, 2.7
**Baseline BMI (kg/m²)**			
18.5–24.9	107	−2.7 (1.0)	−4.6, −0.7
25–29.9	127	−3.3 (1.0)	−5.3, −1.3
≥30	176	−0.7 (0.9)	−2.4, 1.0

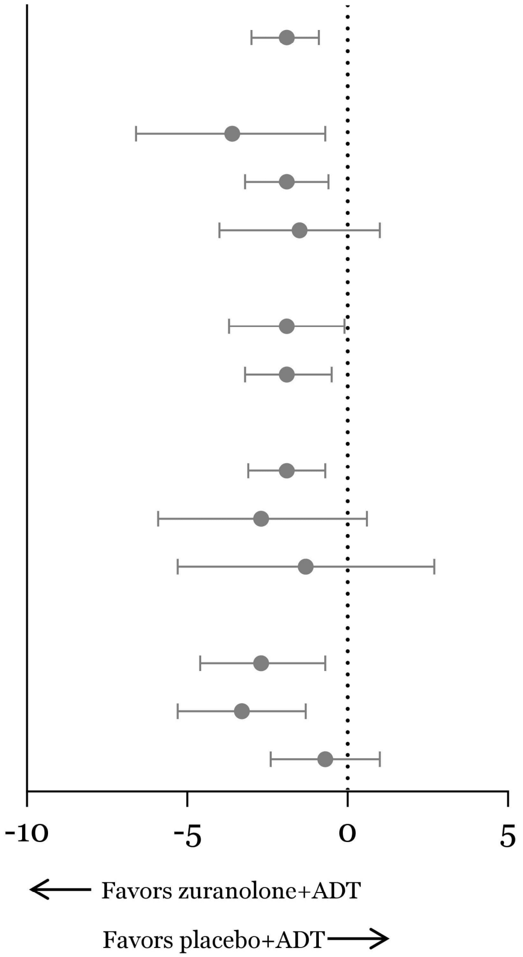

Fig. S1 Forest plot of treatment differences in HAMD-17 total score at Day 3 (full analysis set)

ADT antidepressant therapy, BMI body mass index, CI confidence interval, HAMD-17 17-item Hamilton Rating Scale for Depression, LSM least squares mean, SE standard error.

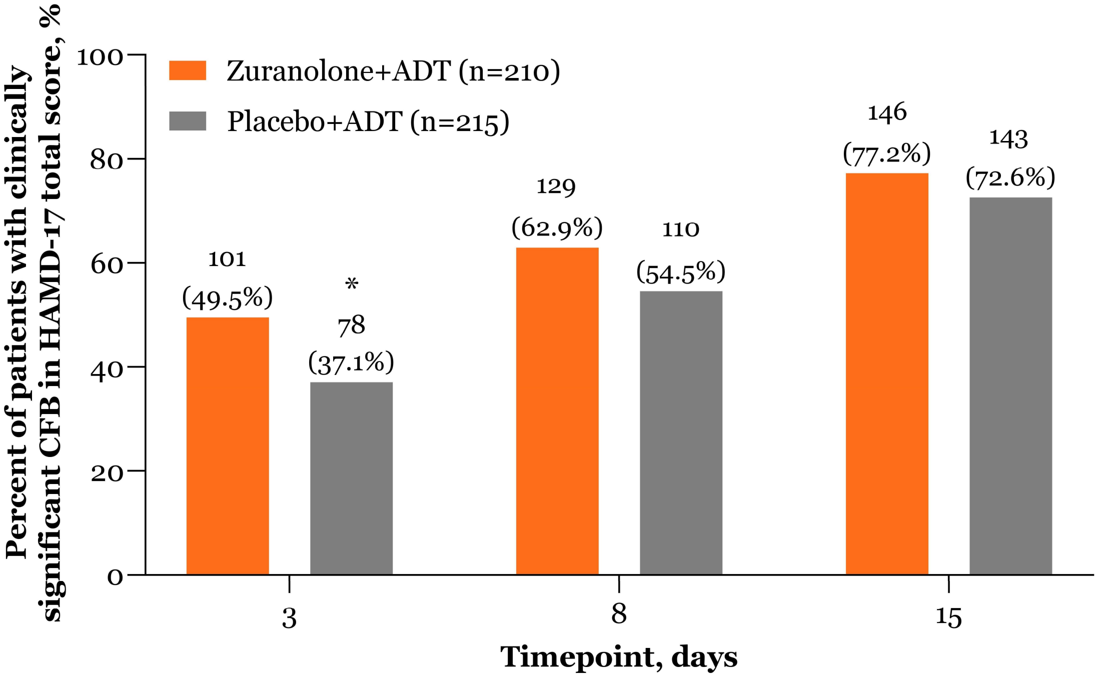

Zuranolone+ADT, n=	204	205	189
Placebo+ADT, n=	210	202	197

Fig. S2 Clinically significant CFB in HAMD-17 total score by time point and treatment group

A clinically significant CFB in HAMD-17 total score as assessed by the MCT was defined as a reduction equal to or greater than −9.0.

Data are shown for the full analysis set. These endpoints were not adjusted for multiplicity, and p-values are considered nominal. *$p<0.05$

ADT antidepressant therapy, CFB change from baseline, HAMD-17 17-item Hamilton Rating Scale for Depression, MCT meaningful change threshold.

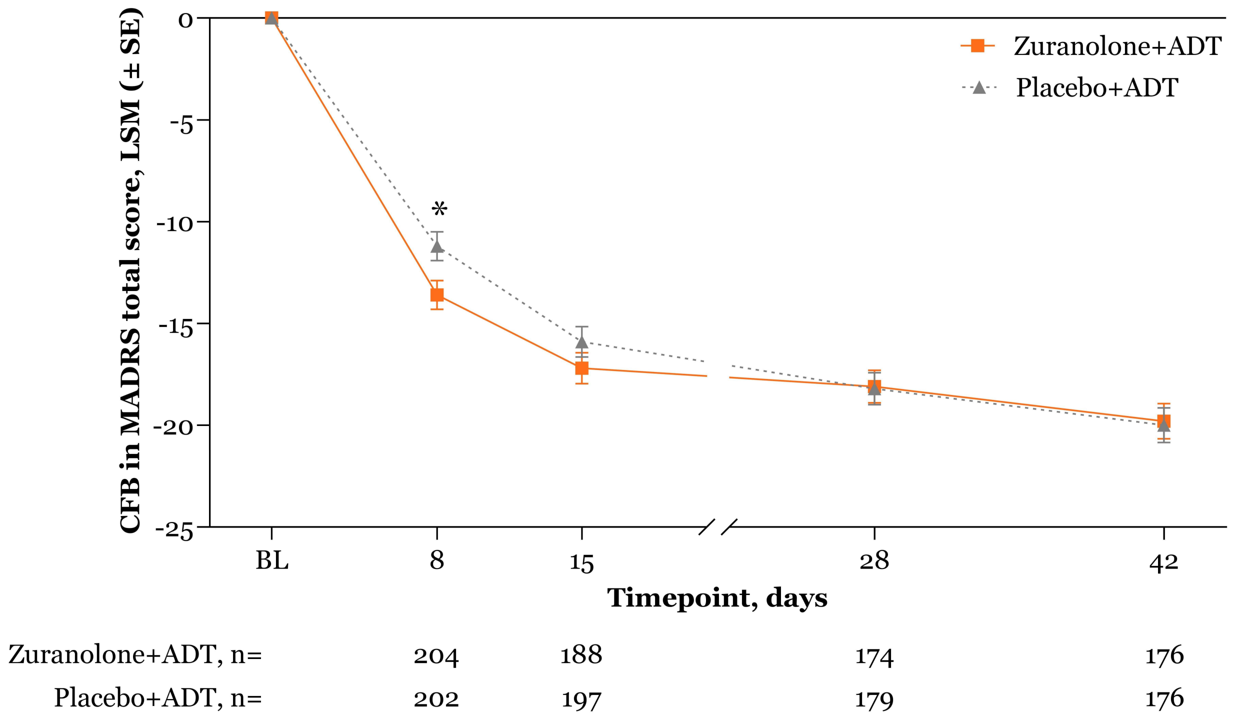

Fig. S3 CFB in MADRS total score

Data are shown for the full analysis set using a mixed effects model for repeated measures. These endpoints were not adjusted for multiplicity, and p-values are considered nominal.

*p<0.05

ADT antidepressant therapy, BL baseline, CFB change from baseline, LSM least squares mean, MADRS Montgomery-Åsberg Depression Rating Scale, SE standard error.

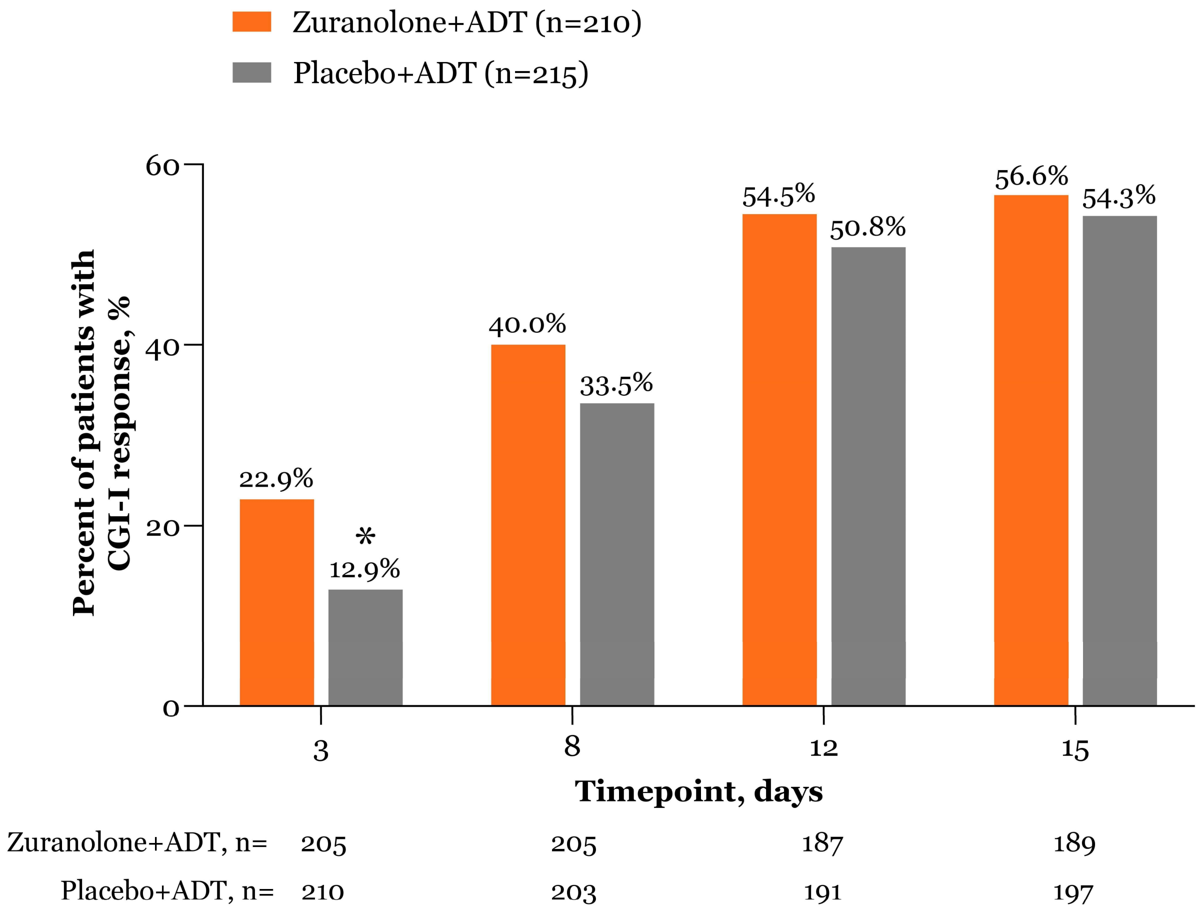

Fig. S4 CGI-I response by study visit

Data are shown for the full analysis set. Response was defined as a CGI-I score of “very much improved” or “much improved.” These endpoints were not adjusted for multiplicity, and p-values are considered nominal. *$p<0.05$

ADT antidepressant therapy, CGI-I Clinical Global Impression-Improvement.
